# Beneficial Effects of Citrus Bergamia Polyphenolic Fraction on Saline Load-Induced Injury in Primary Cerebral Endothelial Cells from the Stroke-Prone Spontaneously Hypertensive Rat Model

**DOI:** 10.3390/nu15061334

**Published:** 2023-03-09

**Authors:** Rosita Stanzione, Maurizio Forte, Maria Cotugno, Francesca Oppedisano, Cristina Carresi, Simona Marchitti, Vincenzo Mollace, Massimo Volpe, Speranza Rubattu

**Affiliations:** 1 IRCCS Neuromed, 86077 Pozzilli, Italy; stanzione@neuromed.it (R.S.); maurizio.forte@neuromed.it (M.F.); maria.cotugno@neuromed.it (M.C.); simona.marchitti@neuromed.it (S.M.); 2 Department of Health Science, Institute of Research for Food Safety & Health IRC-FSH, University Magna Graecia, 88100 Catanzaro, Italy; oppedisanof@libero.it (F.O.); carresi@unicz.it (C.C.); mollace@unicz.it (V.M.); 3 IRCCS San Raffaele, 00163 Rome, Italy; massimo.volpe@uniroma1.it; 4 Department of Clinical and Molecular Medicine, School of Medicine and Psychology, Sapienza University of Rome, 00185 Rome, Italy

**Keywords:** salt loading, endothelial cell, oxidative stress, mitochondrial dysfunction, bergamot, polyphenols, stroke, SHRSP

## Abstract

High salt load is a known noxious stimulus for vascular cells and a risk factor for cardiovascular diseases in both animal models and humans. The stroke-prone spontaneously hypertensive rat (SHRSP) accelerates stroke predisposition upon high-salt dietary feeding. We previously demonstrated that high salt load causes severe injury in primary cerebral endothelial cells isolated from SHRSP. This cellular model offers a unique opportunity to test the impact of substances toward the mechanisms underlying high-salt-induced vascular damage. We tested the effects of a bergamot polyphenolic fraction (BPF) on high-salt-induced injury in SHRSP cerebral endothelial cells. Cells were exposed to 20 mM NaCl for 72 h either in the absence or the presence of BPF. As a result, we confirmed that high salt load increased cellular ROS level, reduced viability, impaired angiogenesis, and caused mitochondrial dysfunction with a significant increase in mitochondrial oxidative stress. The addition of BPF reduced oxidative stress, rescued cell viability and angiogenesis, and recovered mitochondrial function with a significant decrease in mitochondrial oxidative stress. In conclusion, BPF counteracts the key molecular mechanisms underlying high-salt-induced endothelial cell damage. This natural antioxidant substance may represent a valuable adjuvant to treat vascular disorders.

## 1. Introduction

The stroke-prone spontaneously hypertensive rat (SHRSP) represents a suitable animal model for the dissection of the pathogenetic basis of cerebrovascular damage associated with hypertension [1]. The stroke phenotype is accelerated in this model by feeding with a high-salt/low-potassium Japanese-style diet (JD) [2,3], with renal damage preceding stroke occurrence [4]. The stroke-resistant spontaneously hypertensive rat (SHRSR), which represents the strict control of the SHRSP strain, does not develop vascular damage upon the same dietary treatment despite similar blood pressure levels [3]. A genetic linkage analysis demonstrated that few chromosomal loci contributed in a significant manner to the stroke phenotype variance between the two strains [3]. Subsequent investigations targeted to a chromosome 1 locus (*STR1*), explaining 20% of the stroke phenotype variance, highlighted a key role of mitochondrial dysfunction in mediating the high-salt-favored vascular damage of JD-fed SHRSP [5]. In fact, in this experimental condition, a mitochondrial complex I deficiency is induced by inhibition of the Ndufc2 subunit expression, whose gene maps are at the peak of linkage within *STR1*. Ndufc2 is a fundamental subunit to allow for regular assembly and function of the complex I, with consequent regular activity of the oxidative phosphorylation [5]. Subsequent studies revealed that this molecular mechanism also severely alters mitochondrial structure and function in peripheral blood mononuclear cells of healthy subjects once exposed to either high salt or lipopolysaccharides [6]. Most importantly, a decrease in the Ndufc2 subunit also contributes to both juvenile ischemic stroke and myocardial infarction occurrence in humans [5,6,7,8]. Interestingly, isolated primary cerebral endothelial cells (ECs) [6] from SHRSP, once exposed to saline load, show a significant degree of mitochondrial dysfunction, dependent from a decrease in Ndufc2 subunit expression, with a consequent increase in oxidative stress, reduced viability, and increased necrosis [9,10]. Therefore, this in vitro model mimics the in vivo condition quite well and represents a suitable experimental tool for testing the effects of protective molecules in vitro.

Vegetal substances are known for their beneficial vascular properties in both animal models and in humans due to their ability to counteract oxidative stress, inflammation, and mitochondrial dysfunction [11]. In this regard, our previous studies demonstrated the protective role of *Brassica oleracea* sprout extract, based on both anti-inflammatory and antioxidant actions, toward the high-salt-induced vascular injury of SHRSP [12,13]. The latter evidence, showing a remarkable decrease in both renal and cerebrovascular damage, further supported the significant adjuvant role of favorable nutritional components to combat cardiovascular and cerebrovascular diseases. 

Among the emerging substances of natural origin provided of cardiovascular beneficial properties, the bergamot polyphenolic fraction (BPF), that is, the extract of the bergamot fruit (*Citrus bergamia*), is attracting much attention. BPF reduces serum lipid level (low-density lipoprotein cholesterol and triglycerides) and improves metabolic parameters and endothelial function in both animal models and in humans [14,15,16,17,18,19,20,21]. At the cellular level, BPF shows anti-inflammatory and antioxidant properties and can improve mitochondrial bioenergetics, mitochondrial function, and cell metabolism [16,22,23,24]. Interestingly, the protective action of BPF was also related to its ability to restore autophagy [25,26], a process with a fundamental role in cellular, tissue, and organismal homeostasis since it selectively targets dysfunctional organelles and pathogenic proteins [27]. Evidence that BPF can counteract high-salt-favored vascular injury in the context of arterial hypertension is still lacking. 

On these premises, the goal of the present study was to test in vitro the potential beneficial impact of BPF toward high-salt-induced injury and the underlying molecular mechanisms in SHRSP cerebral ECs, as starting knowledge for subsequent in vivo investigations.

## 2. Materials and Methods

### 2.1. Preparation of the Bergamot Polyphenolic Fraction (BPF) 

This step was performed through a standardized and previously reported procedure [14]. In particular, the cultivations of *Citrus bergamia* Risso & Poiteau are present along the Ionian coast of Calabria, in a geographical area of about 90 km between Bianco and Reggio Calabria, Italy. After harvesting, the peeled fruits were squeezed to obtain the bergamot juice, from which the oily fraction was removed by stripping. Furthermore, the juice was clarified by ultrafiltration. At the end of this process, the clarified juice was eluted through a polystyrene resin column, using a KOH solution, to retain the polyphenolic compounds with a molecular weight between 300 and 600 Da. Incubation of the basic eluate on a rocking platform allowed for a reduction in the furocoumarin content, on which the shaking time depended. In the next step, this phytocomplex was neutralized by filtration on cationic resin at acid pH, and after drying under vacuum and mincing, it was transformed into powdered BPF. Analysis of the BPF powder by UHPLC-HRMS/MS determined that it consisted of 40% flavonoids and 60% carbohydrates, fatty acids, pectins, and maltodextrins. The flavonoid profile analyzed by high-resolution mass spectrometry (Orbitrap spectrometer) and HRMSMS (ddMS2, data-dependent MS/MS) includes neoeriocitrin, naringin and neohesperidin. In addition, the entire HMG family is present with bruteridin and melitidin together with flavonoids such as 6.8-di-C-glycosides [24,28,29]. The BPF used in the present study was prepared and characterized by polyphenol content a month before performing the experiments. 

### 2.2. Cell Isolation and Culture

Primary cerebral ECs were isolated from newborn SHRSP rat brains (1–3 days old) by enzymatic and mechanic digestions and subsequent positive selection using microbeads magnetically labeled with CD31 antibody (Miltenyi Biotec, Bergisch Gladbach, Germany). For the enzymatic and mechanic digestions, neural tissue from neonatal brains was dissociated into single-cell suspension by using the Neural Tissue Dissociation Kit (Miltenyi Biotec) and the gentle MACS Dissociator (Miltenyi) with a specific program for neonatal brain. Afterword, the positive selection of ECs with CD31 antibody (Endothelial Cell Isolation Kit, Miltenyi) was performed by a separation over a MACS column placed in the magnetic field of a MACS separator (Miltenyi). ECs were grown in DMEM/F12 medium (Thermo Fisher Scientific, Waltham, MA, USA) supplemented with 5% FBS (Euroclone Srl, Pero, Italy) and ECGS (Sigma Aldrich–Merck, Darmstadt, Germany) on gelatin-precoated dishes at 37 °C and 5% CO_2_ in an humified incubator. Cells were used between passages 1–4 for all experiments, as previously reported [9]. Animal experiments for EC isolation were performed in accordance with the European Commission guidelines (Dlg 2010/63/EU) and the protocol was approved by Italian Ministry of Health (protocol n.: 448/2022-PR).

### 2.3. Immunostaining of Primary Cerebral ECs for CD31

The purity of ECs was confirmed by immunofluorescence for CD31, a transmembrane glycoprotein expressed by ECs. For this purpose, 2 × 10^4^ cells were plated in an 8-chamber slide and fixed for 10 min with 4% PFA, washed in PBS, blocked with 5% goat normal horse serum (Vector Laboratories, Burlingame, CA, USA) and incubated overnight at 4 °C with anti-CD31 antibody (0AAF00819-Aviva System Biology, San Diego, CA, USA). Then, Alexa fluor 488 (Invitrogen Carlsbad, CA, USA) was used for detection in fluorescence. Cell nuclei were stained with Höechst reagent (Thermo Fisher Scientific). Images were randomly taken with a fluorescence microscope.

### 2.4. Cell Treatments

Preliminary experiments testing different BPF concentrations (from 50 to 500 µg/mL) on cell viability identified 250 µg/mL as the appropriate concentration to perform all studies in our cellular model exposed to salt loading. The latter is a noxious stimulus previously used in our studies to mimic the in vivo exposure of SHRSP to JD as a stroke-promoting diet [9,10,30]. Therefore, to test the effects of BPF on cell proliferation, viability, oxidative stress, angiogenesis, wound healing, and mitochondrial function, ECs were exposed for 72 h to the following treatments: NaCl 20 mM, BPF alone (250 µg/mL), and NaCl + BPF (20 mM and 250 µg/mL, respectively). Both NaCl and BPF were diluted in the cell medium. All experiments were performed at least in triplicate. 

### 2.5. Cell Viability

To test cell proliferation and viability, we used the quantitative colorimetric MTT (3-(4,5-Dimethylthiazol-2-yl)-2,5-Diphenyltetrazolium Bromide) test (Sigma Aldrich–Merck). MTT is a yellow powder containing bromide, which in living cells is transformed, by an enzymatic scission, into an insoluble blue/purple precipitate, the formazan. To perform the assay, ECs were plated in a 96-multiwell plate at a density of 1 × 10^4^ cells per well, and they were exposed to 20 mM NaCl for 72 h, as previously described [9,10]. After the treatment, ECs were incubated for 2–3 h with 10 µL of MTT reagent (5 mg/mL) in a 37 °C, 5% CO_2_ incubator. Then, 100 μL of DMSO were added to each well, and the mixture was stirred well until the formazan was completely dissolved. Finally, the absorbance of the solubilized substrate was measured with a microplate reader (Biorad, Hercules, CA, USA) at a wavelength of 570 nm.

### 2.6. Cellular Reactive Oxygen Species (ROS) Measurement

Cellular ROS were evaluated using the fluorescent probe 2′,7′-Dichlorofluorescein diacetate (DCFH-DA Sigma Aldrich). DCHF-DA is an apolar molecule that diffuses easily in cells, where by two successive enzymatic reactions, it is transformed into DCF, a highly fluorescent molecule that is emitted at a wavelength of 532 nm. The oxidation of DCHF to DCF occurs mainly by H_2_O_2_. Therefore, the fluorescence intensity is considered directly proportional to the quantity of H_2_O_2_ produced by the cells. In our experiments, ECs were treated with 200 µL of 10 μM DCFH-DA for 30 min at 37 °C in the darkness. Production of ROS was measured by a microplate reader (Berthold, Bad Wildbad, Germany) at an excitation wavelength of 485 nm and an emission wavelength of 530 nm.

### 2.7. Angiogenesis Assay

The angiogenesis assay was performed by using a Matrigel matrix (Corning, by Sigma Aldrich–Merck). In the specific, 50 microliters of Matrigel matrix were added to each well of a 96-multiwell plate and allowed to solidify for 1 h at 37 °C. After treatment with NaCl and BPF, 1 × 10^4^ ECs were plated on top of the Matrigel layer and incubated for 4 h. Images were taken with EVOS Cell Imaging Systems (Thermo Fisher Scientific) and the number of master junctions was quantified using a specific plugin “Angiogenesis analyzer” of ImageJ software (National Institutes of Health, Bethesda, MD, USA) [31].

### 2.8. Wound-Healing Assay

Cell migration was evaluated by conventional wound-healing assay. For this purpose, 5 × 10^5^ cells were plated on each well of a 24-multiwell plate until confluence was reached. Then, ECs were incubated with 20 mM NaCl for 72 h either in the absence or in the presence of BPF, and the cell monolayers were damaged by manual scratching with a sterile yellow tip. Images were randomly collected at different time points using an inverted microscope (EVOS Cell Imaging Systems, Thermo Fisher Scientific). Percentage of wound closure was calculated according to the following formula:

% wound closure = [Area of the original wound (t0) − Area of the actual wound (t 72 h)]/Area of original wound (t0) × 100.

Wound area was calculated by ImageJ software (version 1.53t).

### 2.9. Assessment of Mitochondrial Membrane Potential Using JC-1 Staining

To determine the mitochondrial membrane potential depolarization by JC-1 reagent (Thermo Fisher Scientific), 3 × 10^4^ cells were plated on each well of a 24-multiwell plate, and later, they were exposed to 20 mM NaCl for 72 h, as previously described [9], either in the absence or in the presence of BPF in a humidified CO_2_ incubator. After 72 h, ECs were incubated with 10 μg/mL JC-1 at 37 °C for 20 min. After incubation, cells were washed twice with ice-cold PBS 1X. Finally, the images were taken with EVOS Cell Imaging Systems (Thermo Fisher Scientific). JC-1 is a cationic dye that exhibits potential-dependent accumulation in mitochondria, indicated by a fluorescence emission shift from green to red. Mitochondrial depolarization is indicated by a decrease in the red/green fluorescence intensity. The red-to-green fluorescence intensity ratio (R:G) was calculated by ImageJ software.

### 2.10. Assessment of Mitochondrial Function

To assess mitochondrial function, we measured mitochondrial complex I activity (assessed as NAD^+^:NADH ratio) and ATP levels with two commercially available kits. To investigate the redox status of the ECs after 72 h of treatment, the concentrations of NADH and NAD^+^ were determined using an NAD^+^/NADH Assay Kit (ABCAM) in accordance with the manufacturer’s instructions. The absorbance values were acquired at 450 nm by a microplate reader (Berthold), and data were analyzed following the manufacturer’s protocol.

ATP levels were assessed with an ATP colorimetric assay (ABCAM), which, by a series of enzymatic reactions, forms a product that is quantified at 570 nm using a microplate reader (Berthold). Finally, data were analyzed following the manufacturer’s protocol.

Mitochondrial ROS level was evaluated by using the MitoSOX™ Mitochondrial Superoxide Indicators (Thermo Fisher Scientific) following the manufacturer’s instructions. The MitoSOX Red reagent specifically reacts with superoxide, but not with ROS and reactive nitrogen species (RNS). To perform this experiment, ECs were plated onto 8-well chambered cell culture slides (Corning by Thermo Fisher Scientific) and treated as reported above. After 72 h of high salt exposure, ECs were treated with MitoSOX Red (5 μM) for 30 min at room temperature and then washed. Cell nuclei were stained by Höechst reagent (Thermo Fisher Scientific). MitoSOX Red fluorescence and Höechst were acquired by a fluorescence microscope Axiophot2 (Zeiss, Oberkochen, Germany), and MitoSOX fluorescent signal was determined with Image J.

### 2.11. Statistical Analysis

All values are expressed as mean ± standard error (SEM). Comparisons between the experimental groups were performed by one-way ANOVA followed by Bonferroni post hoc test. A *p* value of <0.05 was considered significant. GraphPad Prism (Ver 5.01 GraphPad Software, Inc., La Jolla, CA, USA) statistical software was used for the statistical analysis.

## 3. Results

**Figure 1:**
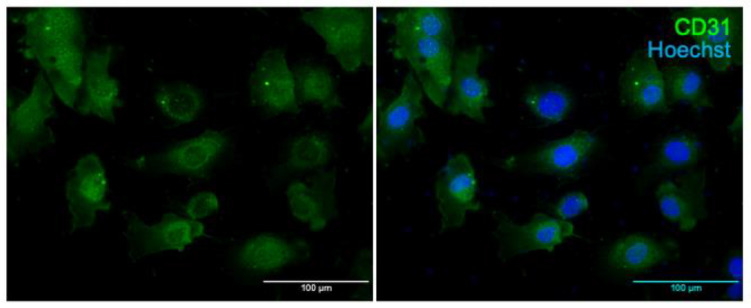
Homogeneity of ECs from brain of neonatal SHRSP. Representative images of immunofluorescence of the isolated cerebral ECs from SHRSP after incubation with CD31 antibody. Alexa Fluor 488 (green) was used as a secondary fluorescent antibody. Cell nuclei were stained with Höechst.

First of all, we checked and confirmed the purity of ECs extracted from the brain of neonatal SHRSP by immunofluorescence for CD31, an established marker of ECs. Results are shown in Figure 1.

**Figure 2:**
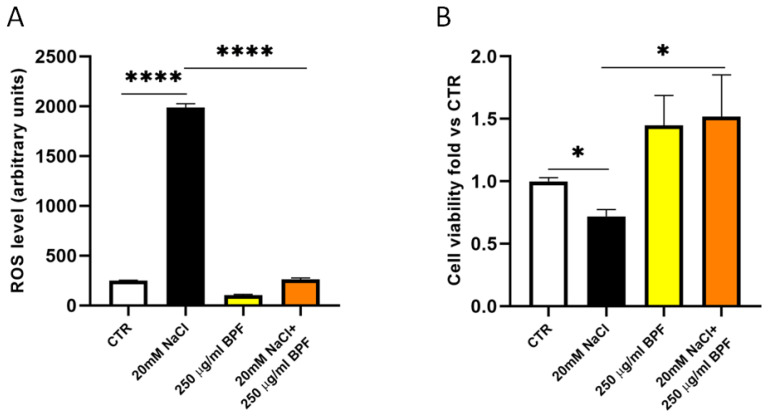
BPF reduces ROS level and rescues cell viability in SHRSP cerebral ECs exposed to high salt load. ECs were treated for 72 h with 20 mM NaCl either in the presence or in the absence of 250 µg/mL BPF. (**A**) Evaluation of total ROS level; (**B**) evaluation of cell viability; N = 3–4. CTR indicates nontreated cells. * *p* < 0.05 and **** *p* < 0.0001 obtained using one-way ANOVA followed by Bonferroni post hoc analysis. Data are reported as mean ± SEM.

**Figure 3:**
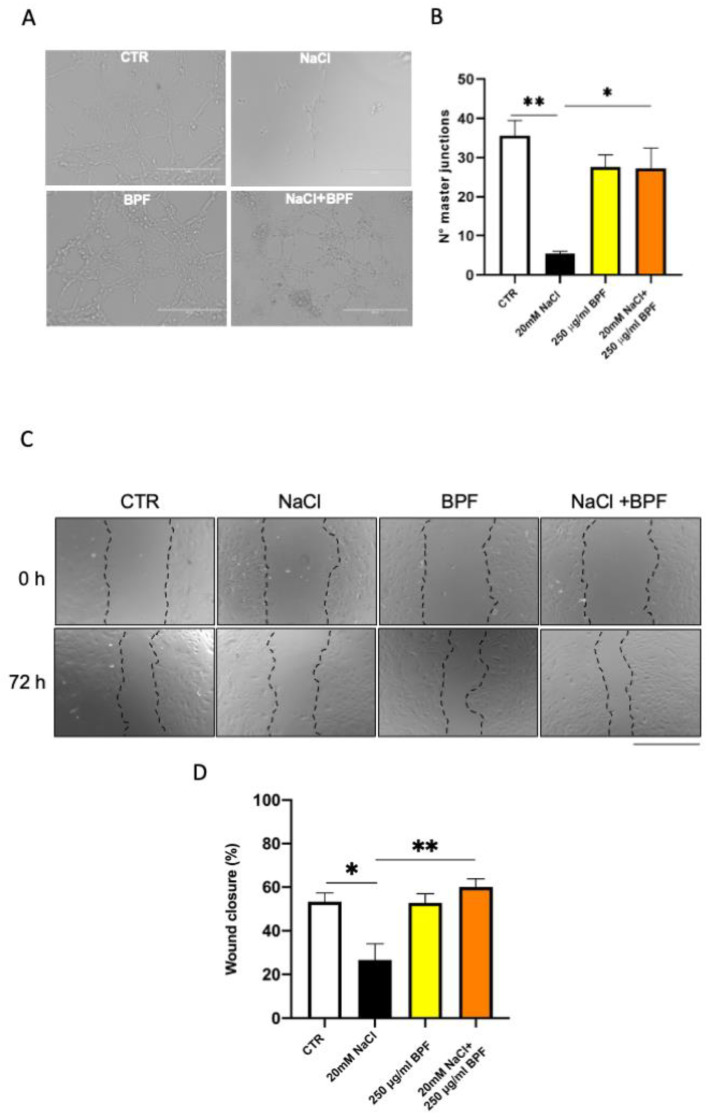
BPF rescues angiogenesis and promotes wound healing in SHRSP cerebral ECs exposed to high salt load. ECs were exposed to 20 mM NaCl for 72 h either in the presence or in the absence of 250 µg/mL BPF. CTR indicates nontreated cells. Representative images of Matrigel assay (**A**) and the quantification of master junctions (**B**); representative images of scratch wound-healing assay (**C**) and relative quantification of wound closure percentage (**D**). N = 3. * *p* < 0.05 and ** *p* < 0.01 obtained using one-way ANOVA followed by Bonferroni post hoc analysis. Data are reported as mean ± SEM.

**Figure 4:**
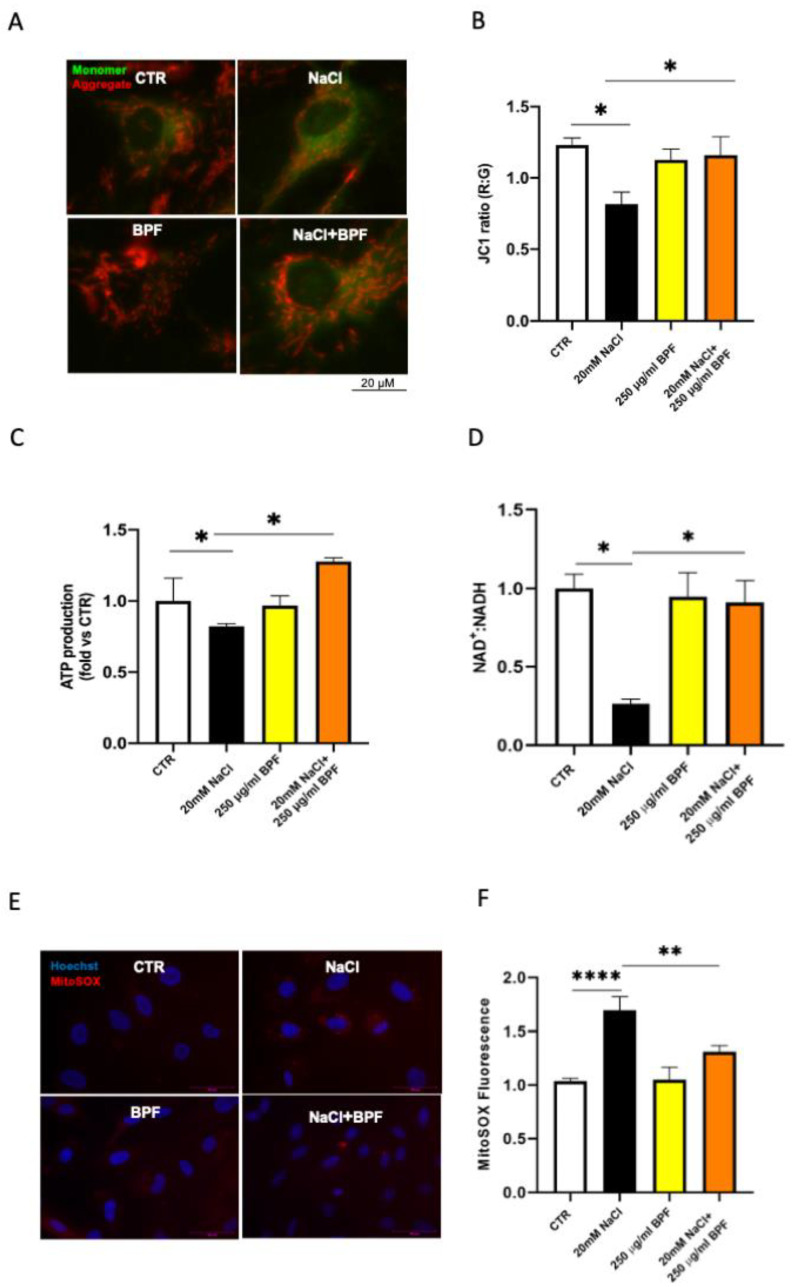
BPF exerts beneficial effect on mitochondrial function in SHRSP cerebral ECs exposed to high salt load. Fluorescence microscope analysis of mitochondrial membrane potential (ΔΨm) levels through JC1 dye; representative images (**A**) and corresponding quantification (**B**) are shown. (**C**) ATP levels, (**D**) NAD^+^:NADH ratio (as a measure of Complex I activity), (**E**,**F**) mitochondrial ROS production detected by MitoSOX fluorescence and its graphical quantification. CTR indicates nontreated cells. N = 3–4. * *p* < 0.05, ** *p* < 0.01 and **** *p* < 0.0001 obtained using one-way ANOVA followed by Bonferroni post hoc analysis. Data are reported as mean ± SEM.

As previously shown [9,10,30], in the current set of studies, we confirmed that the saline load increased oxidative stress; reduced cell proliferation, viability, and migration; impaired angiogenesis, and induced mitochondrial dysfunction with an impairment of mitochondrial membrane potential, reduced complex I activity, and ATP synthesis in SHRSP primary cerebral ECs (Figure 2, Figure 3 and Figure 4). 

In the presence of BPF, cerebral ECs exposed to saline load for 72 h significantly reduced ROS production (Figure 2A) and rescued cell proliferation and viability (Figure 2B). Of note, BPF alone increased cell viability (Figure 2B).

BPF rescued the angiogenetic property of ECs, as shown by the increased number of master junctions in cells exposed to the high-salt treatment in the presence of BPF (Figure 3A,B). Moreover, BPF prompted wound healing by favoring EC migration (Figure 3C,D). In fact, the wound appeared significantly covered by migrated cells upon the combined treatment with salt loading and BPF.

We also tested the impact of BPF on the saline load-induced mitochondrial dysfunction. First, we confirmed that salt loading caused a significant impairment of the mitochondrial membrane potential, which is fundamental to preserve cell integrity. Then, we observed that BPF rescued the mitochondrial membrane potential (Figure 4A,B), as documented by the increased JC1 red-to-green fluorescence ratio. BPF also rescued both ATP synthesis and complex I activity in cerebral ECs under saline load (Figure 4C,D). Consistently, the mitochondrial ROS production decreased in ECs treated with both saline load and BPF (Figure 4E,F).

The whole results of the present investigation indicate that BPF can antagonize the harmful effects induced by high salt load in cerebral ECs, obtained from the SHRSP rat model, on several parameters of cell survival and function.

## 4. Discussion

In the present study we demonstrate that BPF, a vegetal extract obtained from the *Citrus bergamia* fruit, exerts a significant protective effect toward the high-salt-induced injury in primary cerebral ECs isolated from the brain of newborn SHRSP rats. In fact, BPF treatment was able to rescue relevant cellular processes, including proliferation, viability, migration, angiogenesis, and mitochondrial function, which are all compromised upon saline load in the SHRSP experimental model. The latter represents a well-characterized animal model of human disease, particularly for its ability to accelerate cerebrovascular events upon high-salt dietary feeding, which is also based on a genetic predisposition [2,3,4,5,9,12,13,32]. The endothelial dysfunction precedes stroke occurrence in the JD-fed SHRSP as well as in humans [33,34,35,36], an observation that further supports the valuable role of this model for studies on the human disease. Interestingly, as shown in previous studies, the SHRSP primary cerebral ECs exposed to saline load quite closely mimic the in vivo condition and represent a unique experimental tool to test in vitro the effects of protective substances toward the molecular mechanisms involved in the higher predisposition to the vascular damage of the strain [9,10,30].

Herein, based on the available evidence supporting the beneficial properties of BPF in other experimental contexts [14,37,38,39], we aimed to test the potential protective effect of BPF on the cellular parameters known to be compromised upon saline load in the stroke predisposition of the SHRSP strain. We paid particular attention to the ability of BPF to rescue mitochondrial functional parameters (such as mitochondrial membrane potential, ATP synthesis, and complex I activity). A severe mitochondrial dysfunction, dependent on a complex I deficiency, has been previously identified as one of the main pathogenic molecular mechanisms underlying the higher predisposition of the JD-fed SHRSP to vascular damage and its dramatic consequences [5]. In fact, a significant reduction in complex I Ndufc2 subunit expression was observed in the JD-fed SHRSP. Importantly, we have already demonstrated that the recovery of the mitochondrial dysfunction through the correction in complex I deficiency with nicotinamide administration delayed both renal and cerebrovascular damage occurrence in JD-fed SHRSP [9].

The primary cerebral ECs also show an impaired mitochondrial function once exposed to the saline load [9,10,30].

As a result of our present study, BPF exposure led to a significant increase in cell survival, a reduction in oxidative stress and an increase in endothelial cell tubular formation. Remarkably, mitochondrial function was improved with a significant decrease in mitochondrial oxidative stress production.

Our current evidence is consistent with the results of previous investigations and strongly points to BPF as a valuable adjuvant substance to combat cardiovascular diseases. In this regard, an integrated therapeutic strategy, combining standard pharmacological treatments with natural protective substances of either vegetable or animal origin, can reduce the risk of common cardiovascular diseases such as stroke and ischemic heart disease. To support this concept and further validate the suitability of our rat model, we previously reported the significant protective effect of a *Brassica oleracea* sprout extract administration toward renal and cerebrovascular damage occurrence in JD-fed SHRSP [12]. Moreover, the administration of the natural disaccharide trehalose, present in different foods, led to a significant delay in renal damage and stroke occurrence in JD-fed SHRSP [40].

Among the natural available compounds, the bergamot polyphenolic fraction (BPF), extracted from the bergamot fruit (*Citrus bergamia*), a plant endemic to the Calabrian Ionian coast in Southern Italy, belongs to a class of molecules (the polyphenols) that are well known for their protective properties on human health. In particular, bergamot is rich in flavonoid glycosides, such as neoeriocitrin, neohesperidin, and naringin; and glycosylated polyphenols, such as bruteridin and melitidin [28]. Several studies have demonstrated the beneficial effects of polyphenols against widespread pathologies, including cardiovascular diseases, both in preclinical models and in humans [18]. Regarding stroke, a neuroprotective effect was reported even when polyphenols were administered after stroke induction, indicating that these molecules can also contribute to the recovery of patients suffering from stroke [18]. Contrasting effects were observed in schizophrenia [41,42]. The protective functions of BPF are mainly based on antioxidant, anti-inflammatory, lipid-lowering, and hypoglycemic effects of polyphenols [43]. Of note, polyphenols act as both reactive oxygen species scavengers and metal chelators [44]. Moreover, they activate transcription factors such as erythroid 2-related factor 2 (Nrf2), which are able to stimulate the expression of several antioxidant enzymes, including superoxide dismutase (SOD), heme oxygenase-1 (HO-1), catalase, glutathione reductase, and glutathione-S-transferase [45]. In addition, polyphenols exert an anti-inflammatory property that is based on their ability to modulate immune cell regulation, inflammatory gene expression, and the synthesis of inflammatory mediators [46].

In vitro studies revealed that BPF stimulated higher mitochondrial activity with increased ATP production from oxidative metabolism in both isolated mitochondria and porcine aortic endothelial cells [24]. In addition, BPF carried out its beneficial effect on the mitochondrial permeability transition pore (mPTP) phenomenon by desensitizing the pore opening [24], a known molecular mechanism causing cell damage [43]. In fact, BPF inhibited the Ca_2_^+^-activated F_1_F_O_-ATPase, therefore counteracting the opening and size of the mPTP with a final protective effect on mitochondrial dysfunction [24]. In the same cell line, the authors demonstrated that BPF counteracted the toxic effect of doxorubicin on cell viability and mitochondrial function [29]. Based on this in vitro study, BPF, by restoring the correct metabolic cellular functions, can behave as a positive agent toward the cardiovascular disorders resulting from the toxic action of doxorubicin.

Moreover, the protective effects of BPF were reported in a few models of cardiovascular diseases. For instance, consistently with the above-mentioned in vitro data, BPF exerted an antioxidant cardioprotective effect in a rat model of doxorubicin-induced cardiac damage [26]. Interestingly, in this work, the protective action of BPF was related to its ability to restore autophagy. BPF administration in the hyperlipidemic Wistar rat induced a significant reduction in malondialdehyde and glutathione peroxidase serum levels, two known markers of oxidative stress [47].

Furthermore, in a rat model of angioplasty, the pretreatment with bergamot essential oil reduced smooth muscle cell proliferation and neointima formation. This effect was associated with reduced free radical formation and reduced expression of LOX-1, the receptor for oxidized low-density lipoprotein [48].

A human study performed in subjects with moderate hypercholesterolemia evaluated the effects of a Bergamot extract on cardiometabolic parameters, including plasma lipids, atherogenic lipoproteins, and subclinical atherosclerosis, within a relatively short time frame of six months. As a result, the bergamot extracts reduced plasma lipids and improved the lipoprotein profile. Remarkably, a reduced subclinical atherosclerosis (assessed as carotid intimal media thickness) was observed [49].

Altogether, the available evidence, along with the results of the present study, strongly support the role of bergamot toward vascular protection and its potential role as an adjuvant for the treatment of vascular disorders and related acute events.

## 5. Conclusions

We provide the first evidence that BPF is a natural antioxidant able to counteract high-salt-induced injury in a suitable experimental tool, the primary cerebral endothelial cells obtained from the SHRSP model. The latter represents an optimal animal model of human disease, regarding the hypertensive target organ damage favored by high salt exposure. In our study, the treatment with BPF in the presence of high salt, a noxious stimulus, allowed for the recovery of all cellular vital parameters and turned off the key molecular mechanisms underlying endothelial cell damage and dysfunction. This in vitro study represents a fundamental basis for further in vivo investigations testing the impact of BPF toward cerebrovascular accidents. This vegetal substance, as part of the Mediterranean diet, may become an attractive and useful adjuvant to either prevent or treat vascular disorders, such as stroke, associated with hypertension.

## Data Availability

Not applicable.
